# A Variable Clock Underlies Internally Generated Hippocampal Sequences

**DOI:** 10.1523/JNEUROSCI.1120-21.2022

**Published:** 2022-05-04

**Authors:** Xinyi Deng, Shizhe Chen, Marielena Sosa, Mattias P. Karlsson, Xue-Xin Wei, Loren M. Frank

**Affiliations:** ^1^Department of Data Science, Beijing University of Technology, Beijing 100124, People's Republic of China; ^2^Department of Statistics, University of California, Davis, Davis, California 95616; ^3^Center for Integrative Neuroscience and Department of Physiology, University of California, San Francisco, San Francisco, California 94158; ^4^Department of Neuroscience, University of Texas at Austin, Austin, Texas 78751; ^5^Howard Hughes Medical Institute, University of California, San Francisco, San Francisco, California 94158; ^6^Kavli Institute for Fundamental Neuroscience, University of California, San Francisco, San Francisco, California 94158

**Keywords:** flexibility, hippocampus, learning, memory, replay, sharp-wave ripple

## Abstract

Humans have the ability to store and retrieve memories with various degrees of specificity, and recent advances in reinforcement learning have identified benefits to learning when past experience is represented at different levels of temporal abstraction. How this flexibility might be implemented in the brain remains unclear. We analyzed the temporal organization of male rat hippocampal population spiking to identify potential substrates for temporally flexible representations. We examined activity both during locomotion and during memory-associated population events known as sharp-wave ripples (SWRs). We found that spiking during SWRs is rhythmically organized with higher event-to-event variability than spiking during locomotion-associated population events. Decoding analyses using clusterless methods further indicate that a similar spatial experience can be replayed in multiple SWRs, each time with a different rhythmic structure whose periodicity is sampled from a log-normal distribution. This variability increases with experience despite the decline in SWR rates that occurs as environments become more familiar. We hypothesize that the variability in temporal organization of hippocampal spiking provides a mechanism for storing experiences with various degrees of specificity.

**SIGNIFICANCE STATEMENT** One of the most remarkable properties of memory is its flexibility: the brain can retrieve stored representations at varying levels of detail where, for example, we can begin with a memory of an entire extended event and then zoom in on a particular episode. The neural mechanisms that support this flexibility are not understood. Here we show that hippocampal sharp-wave ripples, which mark the times of memory replay and are important for memory storage, have a highly variable temporal structure that is well suited to support the storage of memories at different levels of detail.

## Introduction

The human brain has the remarkable ability to call up memories with different levels of temporal specificity, including experiences that range in extent from seconds to perhaps years. The potential utility of this ability has been demonstrated by a recently proposed theoretical reinforcement learning framework (Levy et al., 2019). This work showed that hierarchical agents that operate at different levels of temporal abstraction by using “multilevel hindsight experience replay” can learn tasks more quickly, both because they can divide the work of learning behaviors among multiple policies and because they can also explore internal representations of the environment at varying levels of temporal resolution. This result highlights the utility of variable temporal abstraction in the theoretical framework of deep reinforcement learning (Schmidhuber, 1992; Sutton et al., 1999; Bakker and Schmidhuber, 2004; Kulkarni et al., 2016; Konidaris, 2019). How the brain might implement this flexibility remains unknown.

A potential mechanism for this flexibility would involve storing experiences with different levels of temporal specificity to facilitate subsequent retrieval at different levels of specificity. To determine whether the brain might engage such a mechanism, we can examine activity patterns in the rodent hippocampus, a structure critical for storing and retrieving memories for spatial experiences (Eichenbaum and Cohen, 2004). As an animal moves through its environment, individual hippocampal neurons (place cells) are active when the animal occupies specific regions of space, known as the place fields of cells (O'Keefe and Dostrovsky, 1971). In the context of this “on-line” state (Buzsáki, 1989, 2002, 2019; Kay and Frank, 2019), a traversal through a given environment results in the sequential activation of a series of these place cells. This sequential activation is modulated in association with the ∼8 Hz theta oscillation, with the result that firing is most prevalent at the trough and least prevalent at the peak of each theta cycle (Buzsáki, 2002). Importantly, the frequency of theta varies across a range of only ∼1 Hz, with higher frequencies associated with higher running speeds (Hinman et al., 2011). Thus, theta can be understood as stable internal clocking mechanism that organizes spiking activity in the hippocampal network (Buzsáki, 2002).

Hippocampal spiking is also organized within each theta cycle. Gamma frequency (∼40 Hz) oscillations often occur together with theta oscillations in the hippocampal local field potential (LFP) and these gamma oscillations modulate hippocampal spiking as well (Bragin et al., 1995; Lisman and Buzsáki, 2008; Colgin et al., 2009; Kemere et al., 2013; Lisman and Jensen, 2013; Lopes-Dos-Santos et al., 2018). This modulation has been proposed to help maintain a further level of organization of spiking within each theta cycle that is known as a “theta sequence” (Lisman, 2005): within each cycle, sets of cells with overlapping fields will typically fire in a compressed sequence that often recapitulates the longer timescale sequential activity associated with the serial order of the place fields (Skaggs et al., 1996; Foster and Wilson, 2006; Colgin et al., 2009; Gupta et al., 2012).

Time-compressed versions of these sequences are also seen during hippocampal sharp-wave ripple (SWR) events (Lee and Wilson, 2002; Foster and Wilson, 2006; Davidson et al., 2009; Karlsson and Frank, 2009). This replay serves as a mechanism for the reinstatement of previously stored representations (e.g., retrieval) that is thought to promote the longer-term storage and updating of these representations in distributed cortical networks (consolidation; Buzsáki, 2015; Joo and Frank, 2018; Gillespie et al., 2021).

Could SWRs support the storage of memories in a way that would allow for temporally flexible retrieval? If so, then we might expect the temporal structure of these events to vary from event to event. SWRs are named in part because of the associated high-frequency (150–250 Hz) ripple oscillation, but this oscillation is generated locally in hippocampal area CA1 and does not coordinate the replay of experience across the hippocampal network (Sirota et al., 2003; Carr et al., 2012). This coordination may instead be mediated by a slower process. Consistent with that possibility, during SWRs there are transient increases in slow-gamma (20–50 Hz) power and synchrony across dorsal CA3 and CA1 networks of both hemispheres (Carr et al., 2012; Gillespie et al., 2016; Oliva et al., 2018). The slow-gamma phase also provides a reliable internal temporal organization (“clock”) for replay events (Carr et al., 2012), and it has been suggested that these gamma oscillations govern the temporal segmentation of spatial content in the following replay sequences: during phases of high neural activity within the gamma cycle, spatial representations are often focused on a single location, whereas during phases of low neural activity, the spatial representation is more likely to move to adjacent locations (Pfeiffer and Foster, 2015).

Slow gamma varies over a wide range, but precisely how this variation manifests in hippocampal network during SWRs has not been examined. Moreover, whether this variability is related to the representational content of SWRs is also unknown. To address these issues, we examined the temporal organization of population-spiking events on an event-to-event basis during locomotion and awake immobility as rats learned to perform a memory-guided task. We found that population spiking underlying SWR events had rhythmic structure and exhibited much higher event-to-event variability in periodicity than locomotion-associated spiking sequences. Events with varying periodicity could represent similar spatial experiences, and, surprisingly, variability increased rather than decreased as the environment became more familiar. We hypothesize that this experience-dependent variability in SWR rhythmic organization supports memory storage in support of temporally flexible memory retrieval.

## Materials and Methods

### Subjects, neural recording, and behavioral task

The experimental methods are described in detail in the studies by Karlsson and Frank (2008, 2009) and Kay et al. (2016). In brief, data were taken from four male Long–Evans rats (weight range, 500–600 g) that had been implanted with a microdrive array containing between 14 and 30 independently movable tetrodes, respectively, targeting CA1 and CA3. Tetrodes that never yielded clusterable units across the recordings (11, 10, 8, and 12 d for animals 1–4) were excluded from analysis. Following histologic verification, tetrodes that ended up in areas other than CA1 and CA3 were excluded from analysis. In the analyses presented here, multiunit activity (MUA) from 16, 18, 14, or 17 simultaneously recorded tetrodes, respectively, were included. Unsorted spikes with peak-to-trough width of <0.35 ms were identified as from putative inhibitory interneurons, and unsorted spikes with peak-to-trough width of >0.35 ms were identified as from putative pyramidal neurons.

The MUA recorded from one tetrode was defined as all detectable spike waveforms that cross a minimum amplitude threshold, usually set between 40 and 100 mV. Through standard manual clustering techniques, between 0 and 10 well isolated units were extracted per tetrode in the dataset described here. An average of 23, 53, 30, and 45 single units across all recording epochs, respectively, were clustered. Importantly, the activity of these sorted putative units includes a minority of detectable spikes; an average of 92.94, 89.13, 94.80, and 91.12% of spikes across all recording epochs, respectively, remain “unclassified” because they could not be confidently assigned to a single unit. Thus, a large fraction of the data collected in these recordings would not be used for standard decoding analyses. This exclusion of the majority of the spiking event motivated our use of clusterless decoding analyses (see below).

**Figure 1.**
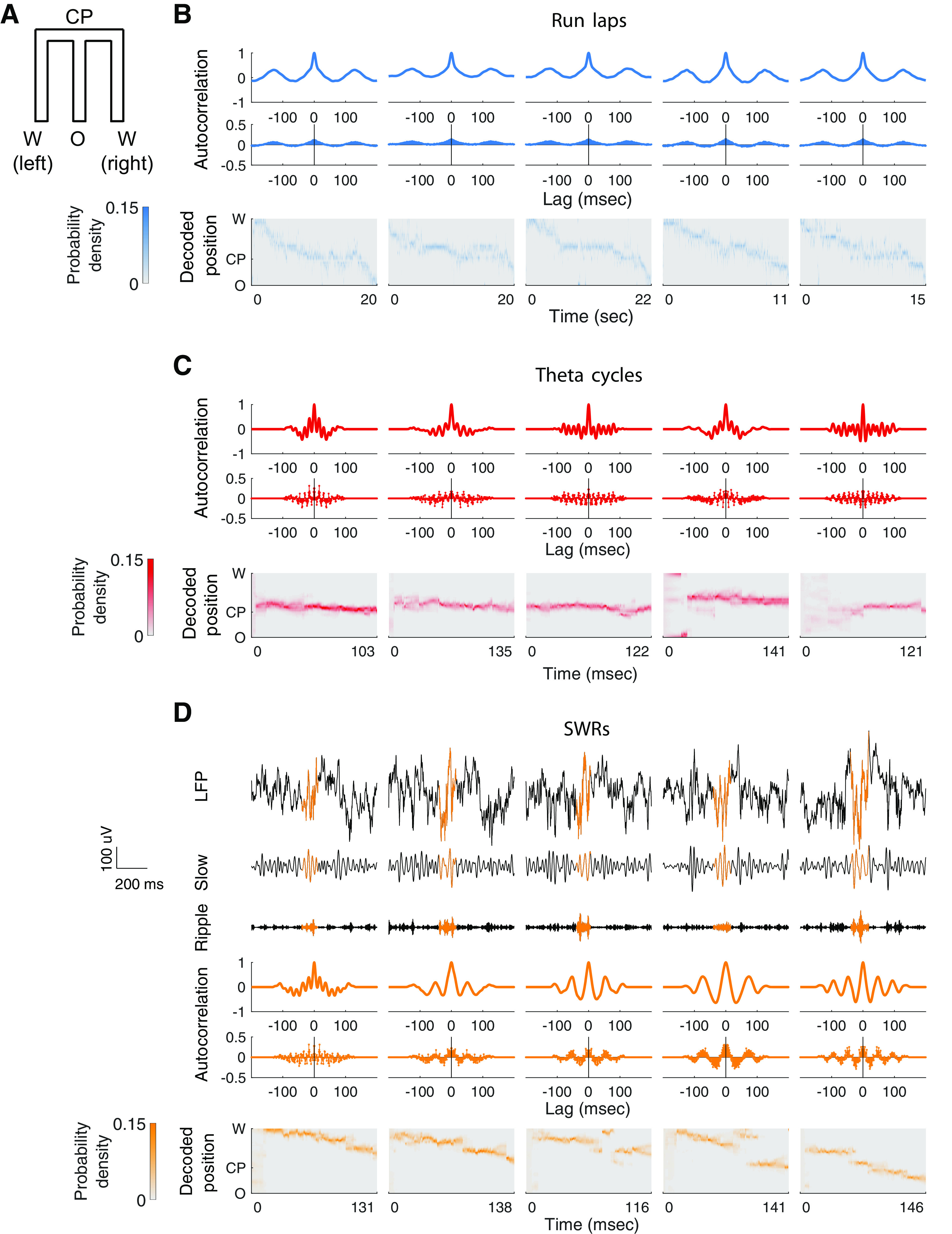
SWR-associated temporal organization of population spiking is rhythmic and exhibits higher event-to-event variability than locomotion-associated population spiking. ***A***, 2D representation of the W-maze. ***B***, Rhythmicity of spiking across traversals of an inbound (W → O) trajectory in the W-maze (run laps) from Animal 2. Each column represents one run lap. Top, Peak-normalized Gaussian-smoothed autocorrelation function of population spiking. Middle, Autocorrelation function of population spiking. Bottom, Decoded trajectory where the heat plot shows the estimated posterior density at each time step. Theta (∼125 ms) modulation of autocorrelation is clearly visible across different laps. ***C***, Rhythmicity of spiking across theta cycles, with rows as in ***B***, taken from inbound runs on Animal 2. Each column represents one theta cycle. Population spiking within each theta cycle was also rhythmically organized with a periodicity within the gamma range (mean period, ∼18 ms). ***D***, Rhythmicity of spiking across SWR events from an example epoch of Animal 2. SWRs were chosen to illustrate inbound spatial content. Each column represents one SWR. Top three rows, Raw and filtered LFP signals from one CA1 tetrode for a corresponding SWR event (from first to third row: raw LFP trace; slow-gamma band, 20–50 Hz; ripple band, 150–250 Hz; the colored highlighted area of each LFP trace denotes the entire duration of the associated SWR event). The bottom three rows are as in ***B***.

The hippocampal data in this article were collected as animals learned to perform a continuous alternation task on a W-shaped maze (76 × 76 cm with 7-cm-wide track; Fig. 1*A*) for liquid reward (condensed milk). Each task epoch lasted, on average, for 15 min. The animal was rewarded each time it visited the end of an arm in the correct sequence. On each “outbound trial,” the animal would start at the food well in position O (“origin”) and run toward the intersection, or position CP (“choice point”), at the top of the center stem, where a choice would need to be made. The correct choice is to alternate between left and right on successive outbound trials. If a correct choice is made, for example, to turn right, the animal would continue to run toward position W(right) (“right food well”) to receive a reward, and then return to the center well at position O (“inbound trial”) to move on to the next outbound trial. If an error is made, the rat is not given a reward and must return to the center well to initiate the next trial. All animal procedures and surgery were reviewed and approved by the University of California, San Francisco, Institutional Animal Care and Use Committee and were in accordance with National Institutes of Health guidelines.

We linearized the actual two-dimensional coordinate position of the rat to a single coordinate. The one-dimensional coordinate indicates the total distance from the center well (position O) in centimeters, with negative numbers indicating trajectories that include a left turn and positive numbers indicating trajectories that include a right turn. When the rat was on the center arm of the maze, the region to which its position was mapped was determined by the direction from which the rat came during inbound trajectories and by the direction it would turn next when it reached the choice point (position CP) during outbound trajectories. Throughout this report, to facilitate ease of visualization, when plotting we label the linearized maze at position O, CP, or W (left or right) instead of the corresponding signed one-dimensional coordinate.

### SWR detection

We performed ripple detection using all tetrodes targeting the CA1 region. SWRs were detected on 4, 11, 7, or 5 tetrodes located in from CA1, respectively, by using an aggregated measure of the root mean square (rms) power in the 150–250 Hz band across the tetrodes (Nádasdy et al., 1999). The aggregated rms power was then smoothed with a kernel (4 ms SD), and SWR events were detected as lasting at least 15 ms >2 SDs of the mean. The entire SWR time was then set to include times immediately before and after the power exceeded the mean. SWR events were further restricted to those that occurred while the animal was moving <4 cm/s. Recording epochs with <50 SWR events were excluded from analysis; under this criterion, 3, 0, 0, and 2 epochs, respectively, were excluded.

### Experimental design and statistical analyses

All analyses were conducted using custom software written in MATLAB (MathWorks).

#### Rhythmic organization of population spiking underlying individual SWRs.

**Figure 2.**
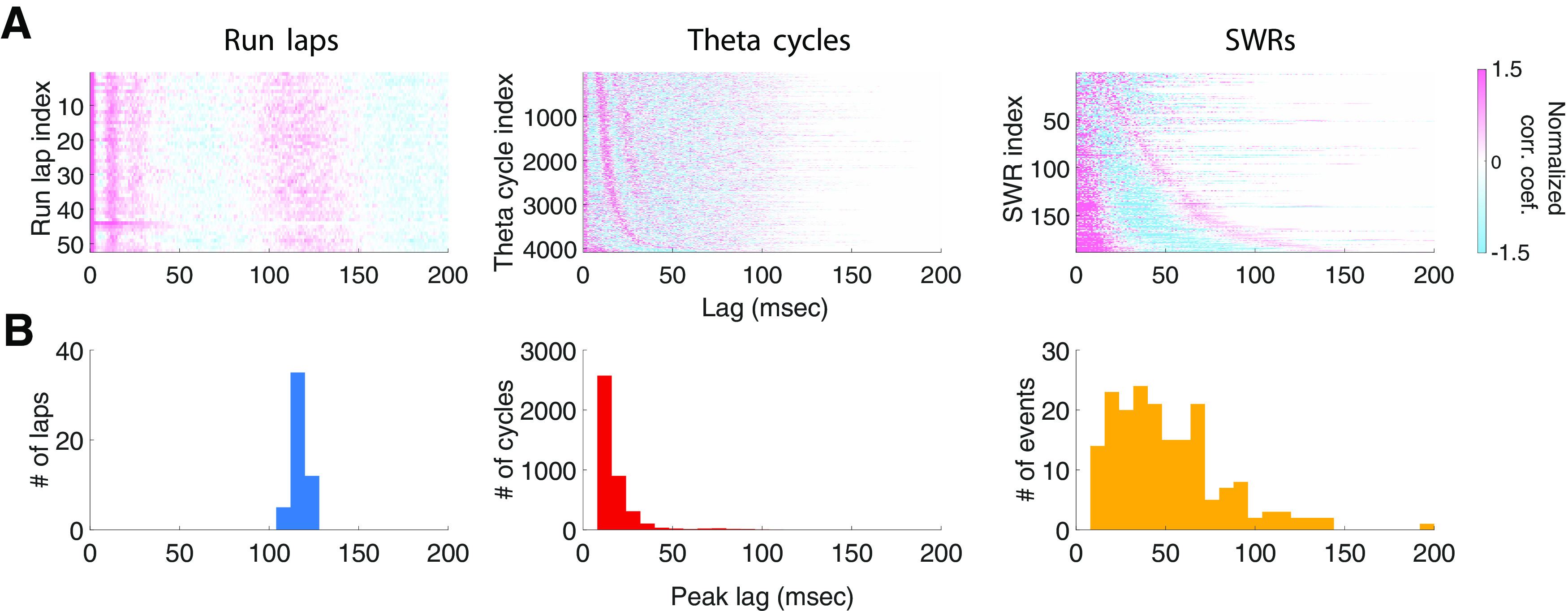
Rhythmic spiking organization of spiking across event types. ***A***, Normalized autocorrelations of multiunit spiking activity during individual run laps (left), theta cycles (middle), and SWR events (right) from an example epoch of Animal 3, sorted in ascending order by the timing of the first positive side peak lag. Every horizontal line is the autocorrelation function of an individual lap, theta cycle, or SWR, respectively; only non-negative lags are displayed. Positive and negative correlation coefficients are plotted in shades of red and blue, respectively. ***B***, Histogram of the timing of the first positive side peak lag of the multiunit spike autocorrelation lags across run laps (left), theta cycles (middle), or SWR events (right), respectively, for an example epoch.

The temporal organization of population spiking underlying individual SWR event was defined using multiunit spiking activity. The population spiking activity was calculated by summing up unsorted spikes recorded on tetrodes that had yielded clusterable units across all days of experiments. We then computed the autocorrelation function of this summed spike train binned at 1 ms with lags up to 200 ms. When sorting spike autocorrelation across events (Fig. 2), we first smoothed the autocorrelation function with a 20 ms Gaussian kernel and then calculated the lag of the first positive side peak.

#### Rhythmic organization of population spiking underlying individual run laps.

The temporal organization of population spiking underlying the individual run lap was defined using multiunit spiking activity. The population spiking activity was calculated by summing up unsorted spikes recorded on tetrodes that had yielded clusterable units across all days of experiments. We then computed the autocorrelation of this summed spike train binned by 1 ms with lags up to 400 ms. When sorting spike autocorrelation across events (Fig. 2), we first smoothed the autocorrelation function with a 100 ms Gaussian kernel and then calculated the lag of the first positive side peak.

#### Rhythmic organization of population spiking underlying individual theta cycles.

We first defined theta cycles as follows: LFP was filtered at 5–11 Hz. Peaks and troughs of the filtered LFP were detected and used to define half-cycles by linear interpolation. Theta cycles were identified as individual cycles whose duration was consistent with the 5–11 Hz frequency range [<200 ms (5 Hz), >90 ms (11 Hz)]. The temporal organization of population spiking underlying individual theta cycles was defined using multiunit spiking activity, similar to SWRs: the population spiking activity was calculated by summing up unsorted spikes recorded on tetrodes that had yielded clusterable units across all days of experiments. We then computed the autocorrelation function of this summed spike train binned by 1 ms with lags up to 200 ms. When sorting spike autocorrelation across events (Fig. 2), we first smoothed the autocorrelation function with a 15 ms Gaussian moving kernel and then calculated the lag of the first positive side peak.

#### Parameter estimation for the distributional fit.

We first computed the histogram of the timings of the first positive side peak lag of SWR-associated population spike autocorrelations across events. We then fit log-normal distribution to this histogram whose parameters, μ and σ, were estimated with maximum likelihood.

#### Clusterless decoding of representational content.

We decoded the spatial representation content of an individual SWR event using a clusterless decoding method (Deng et al., 2015) that does not require multiunit spiking waveforms to be sorted into single units and instead incorporates waveform information of unsorted spikes, by using the theory of marked point process. Briefly, any point process representing neural spiking can be fully characterized by its conditional intensity function. A conditional intensity function describes the instantaneous probability of observing a spike, given previous spiking history. By relating the conditional intensity to specific biological and behavioral signals, we can specify a spike train encoding model. The conditional intensity also generalizes to the marked case, in which a random vector, termed a mark, is attached to each point. Here we use the mark to characterize features of the spike waveform. In the case of tetrode recordings, the mark used was a length-four vector of the maximum amplitudes on each of the four electrodes at every spike time.

Briefly, we characterizes the instantaneous probability of observing a spike with mark $\vec{m}$ at time *t* as a function of some underlying internal state variable *x*(*t*), such as the location of an animal in space that varies across time, using the joint mark intensity function $\lambda (t,\vec{m}|H_{t})$, where *H_t_* is the history of the spiking activity up to time *t*, as follows:

(1) $$\begin{array}{l} \;\lambda (t,\vec{m}|H_{t})\\ =\underset{\Delta \to 0}{\lim}\frac{\text{Pr(a spike with mark vector }\vec{m}\text{ in }(t,t+\Delta ]|H_{t})}{\Delta }\\ =g(x(t),\vec{m}|H_{t}). \end{array}$$

Our decoding algorithm, using discrete-time state-space adaptive filters, computes, at each time point, the un-normalized posterior distribution of the state variable given observed marked spiking activity, as follows:

(2) $$\;p(x_{k}|\Delta N_{k},{\vec{m}}_{k},H_{k})\propto p(\Delta N_{k},{\vec{m}}_{k}|x_{k},H_{k})\;\cdot \int p(x_{k}|x_{k-1})p(x_{k-1}|\Delta N_{k-1},{\vec{m}}_{k-1},H_{k-1})\text{d}x_{k-1}.$$

The $p(\Delta N_{k},{\vec{m}}_{k}|x_{k},H_{k})$ term is the likelihood or observation distribution at the current time, as follows:

(3) $$\begin{array}{l} \;p(\Delta N_{k},{\vec{m}}_{k}|x_{k},H_{k})\\ \propto \{\begin{array}{ll} \exp[-\Delta_{k}\Lambda (t_{k}|H_{k})], & \Delta N_{k}=0;\\ \prod_{i=1}^{\Delta N_{k}}[\lambda (t_{k},{\vec{m}}_{k_{i}}|H_{k})\Delta_{k}]\exp[-\Delta_{k}\Lambda (t_{k}|H_{k})], & \Delta N_{k}>0. \end{array} \end{array}$$

The $\prod_{i=1}^{\Delta N_{k}}[\lambda (t_{k},{\vec{m}}_{k_{i}}|H_{k})\Delta_{k}]$ term characterizes the distribution of firing Δ*N_k_* spikes, such that the mark value of the *i*th spike in the interval $\left(t_{k-1},t_{k}\right]$ is $m_{k_{i}}$, where *i* = 1,…, Δ*N_k_*. The probability of observing a spike regardless of the mark values is denoted by $\Lambda (t|H_{t})=\int_{M}\lambda (t,\vec{m}|H_{t})\text{d}\vec{m}$.

#### Classification of representational content.

We extended the state variable in the clusterless decoder to jointly include a discrete decision state, *I*, which identifies whether each SWR event represents an outbound or inbound trajectory as well as whether the temporal order of activity occurs forward or backward in time (Deng et al., 2016). In our analyses, *I* is an indicator function for a replay event being one of the following four categories: “outbound, forward,” “outbound, reverse,” “inbound, forward,” or “inbound, reverse.” We categorized the representational content of example replay events using a marked point process filter with the joint state variable *x*(*t*), *I*. Finally, if the posterior probability of the decision state Pr(*I*) of an individual SWR event first passes a threshold of 98% for 10 consecutive temporal bins for a particular category of *I*, we assigned the event to that category. SWR events whose decision state probabilities did not pass the threshold and whose representational content cannot be classified into one of the four categories were assigned to a fifth category as unclassified.

#### SWR-triggered spectrogram and local field frequency measures.

SWR-triggered spectrograms were computed using the multitaper method and a 100 ms sliding windows with a 10 ms step size. A *z* score was computed for each frequency band using the mean and SD of the power calculated across the entire behavioral session for each tetrode. For each 100 ms bin, we obtained a normalized measure of power for each frequency band (sharp wave, <20 Hz; slow gamma, 20–50 Hz; ripple, 150–250 Hz) in units of SD from the mean. To quantify gamma-phase locking during SWRs, the phase of coherence for the gamma band was averaged across all CA3–CA1 tetrode pairs for each SWR. Thus, each SWR contributed a single value for each 100 ms temporal bin relative to SWR detection. We combined values across SWRs to obtain a distribution of gamma-phase offsets in each bin. The angular variance of this distribution was taken as a measure of phase locking for each epoch.

#### Hypothesis testing.

Bootstrap methods (1000 iterations) were used to test the homogeneity of variances between peak lags of two behavioral states, Wilcoxon rank-sum tests were used when comparing two groups of peak lags of spike autocorrelation within the same epoch, Kruskal–Wallis tests were used when comparing across multiple groups, and Student's *t* tests were used when comparing the mean of a variable across all lags with zero. Across all epochs for an individual animal, a summary of *p*-values was reported either in text or in supplementary figures. Rayleigh's test for detecting unimodal deviation from circular uniformity was used to evaluate the phase locking of CA3 multiunit spiking to CA1 slow gamma. To evaluate the effects of multiple covariates on the peak lag of the spike autocorrelation across all epochs for an individual animal, linear mixed-effects models were used to account for random effects of epoch. Student's *t* tests were used to estimate the statistical significance of individual fixed-effects coefficients. To provide more interpretable values, standardized coefficients for discrete independent covariates were computed by centering; standardized coefficients for continuous independent covariates are computed by first centering and then dividing by 2 SDs (Gelman, 2008).

## Results

### Event-to-event variability in population spiking structure

To examine the temporal organization of hippocampal population spiking, we analyzed previously recorded electrophysiological data from four male Long–Evans rats implanted with a microdrive array containing multiple independently movable tetrodes targeting CA1 and CA3 (Karlsson and Frank, 2008, 2009; Kay et al., 2016). Data were collected as animals learned to perform a memory-guided alternation task in a W-shaped environment (Fig. 1*A*). The animal was rewarded each time it visited the end of an arm in the correct sequence, starting in the center and then alternating visits to each outer arm and returning to the center (see Materials and Methods).

Our first goal was to identify regularities in the timing of spiking during locomotion and awake immobility. The standard approach, using “clustered” spikes that have been assigned to individual single units, discards the much larger number of spikes that cannot be confidently assigned to a single unit, and the resulting sparse data may not be sufficient for inferring temporal structure. As such, we used the unclustered multiunit spiking data collected across all CA1 and CA3 tetrodes for our analyses (Kloosterman et al., 2014; Deng et al., 2015).

The rhythmicity of the hippocampal spiking activity during a specific period of time was determined by calculating the autocorrelation function of the multiunit spiking activity. Specifically, the “clock speed” for each event was defined as the lag of the first positive side peak of the autocorrelation function using only spikes within the event. This reflects the average time between troughs (or peaks) of spiking activity. Note that clock speed and the timing of the first positive peak lag of the SWR autocorrelation function are inversely correlated, with a lower value of peak lag denoting faster clock speed. We also note that focusing on the structure of the autocorrelation helps avoid challenges associated with the superposition of oscillations with different phases in LFP analyses. In parallel, we decoded the spatial representation expressed during those times using a clusterless decoding method (see Materials and Methods; Deng et al., 2015) that does not require multiunit spiking waveforms to be sorted into single units and instead incorporates waveform information from all spikes.

We first computed the autocorrelation function of hippocampal population spiking during individual runs from one reward site to another (laps) on the track (Fig. 1*B*, five example inbound laps). As expected, the autocorrelation function had a peak at the stereotypical theta lag of ∼125 ms. We then computed the autocorrelation function of hippocampal population spiking during individual theta cycles (for the cycle detection approach, see Materials and Methods). Once again, as expected, population activity within each theta cycle was rhythmically organized with a periodicity within the gamma range (Fig. 1*C*; Lisman, 2005; Lisman and Buzsáki, 2008; Colgin et al., 2009; Lisman and Jensen, 2013; Lopes-Dos-Santos et al., 2018).

Finally, we computed the autocorrelation function of hippocampal population spiking during each SWR event, considering only spikes within the extent of that event. We observed that population spiking activity during individual SWRs is strikingly rhythmically modulated (Fig. 1*D*), consistent with previous demonstrations that SWR events include slow-gamma oscillations that modulate spiking intensity (Carr et al., 2012; Pfeiffer and Foster, 2015). We also observed that the SWR-associated spiking organization appeared to exhibit very high event-to-event variability, even across replay events that express similar spatial representations.

#### The periodicity of SWR-associated spiking follows an approximate log-normal distribution

To examine the temporal structure of spiking across both locomotion-associated and SWR-associated hippocampal population events, we plotted the autocorrelation functions of spiking activity of all hippocampal spiking events within each recording epoch and ordered the events by the timings of the first positive peak lag of the autocorrelation function (Fig. 2*A*).

**Figure 3.**
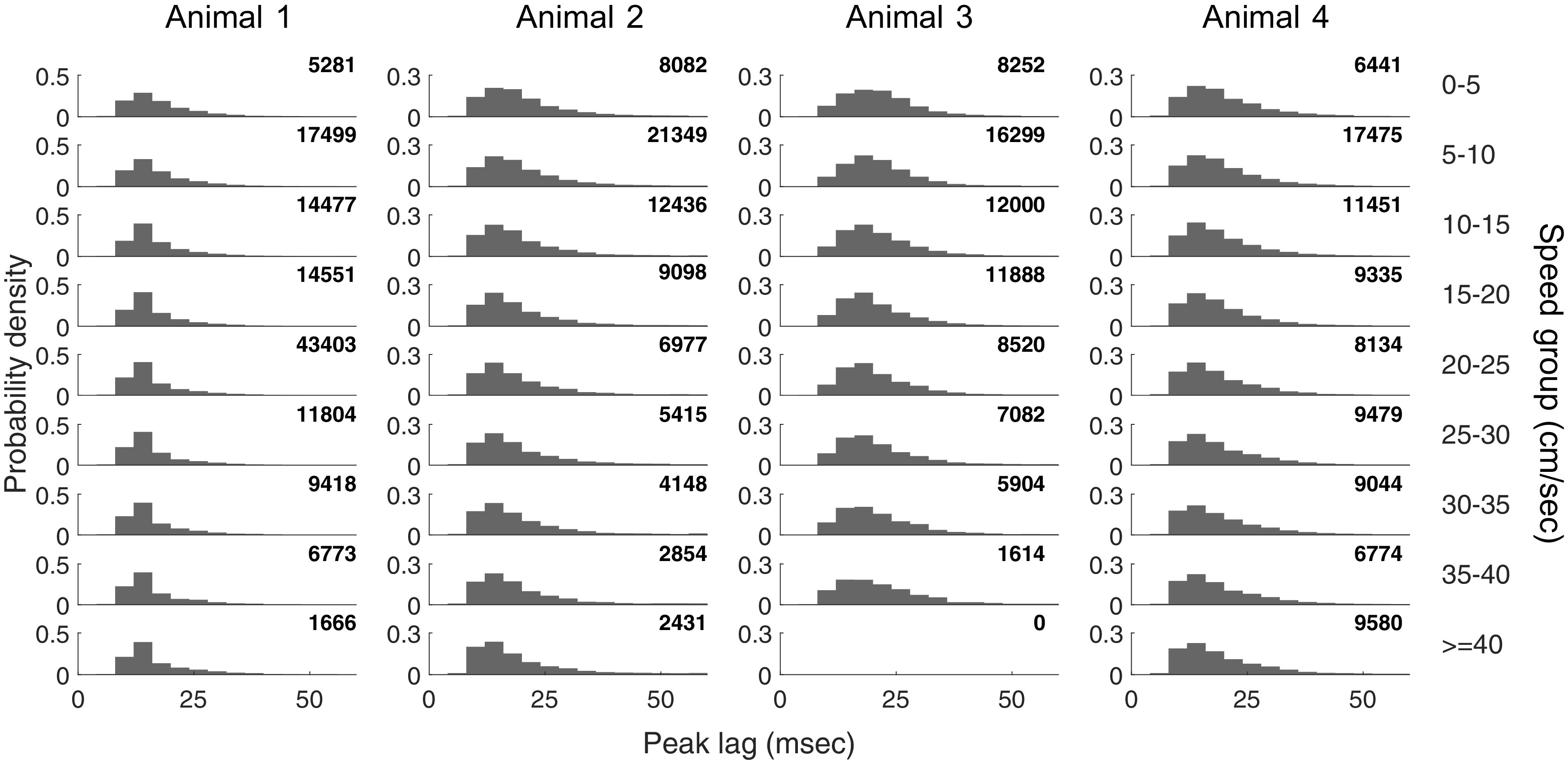
Theta cycles occurring at lower speeds are associated with larger mode values of the empirical distributions. The panel at the intersection of each row and column plots the relative frequency histogram of the first side peak of the theta cycle spike autocorrelation within a speed group for an example animal. The number in the top left of each plot is the number of theta cycles for that animal and speed group. Correlation coefficient (and corresponding *p*-value) between the mode of the empirical distribution and speed: Animal 1, −0.80 (0.0092); Animal 2, −0.95 (0.0001); Animal 3, −0.93 (0.0003); Animal 4, −0.94 (0.0002).

As expected, the periodicity of the spiking seen during run laps clusters tightly around ∼125 ms, consistent with the ∼8 Hz theta (Fig. 2*B*, left), while periodicity for theta cycles clusters tightly at ∼18 ms, consistent with the high end of the typical ∼20–55 Hz slow-gamma range (Fig. 2*B*, middle), respectively. We also observed that, when grouped by movement speed, theta cycles with lower speeds were more likely to be associated with larger mode values of the empirical distributions [Fig. 3; correlation coefficient (and corresponding *p*-value) between the mode of the empirical distribution and speed: animal 1, −0.80 (0.0092); animal 2, −0.95 (0.0001); animal 3, −0.93 (0.0003); animal 4, −0.94 (0.0002)], consistent with an increased prevalence of slow gamma at lower movement speeds (Kemere et al., 2013).

**Figure 4.**
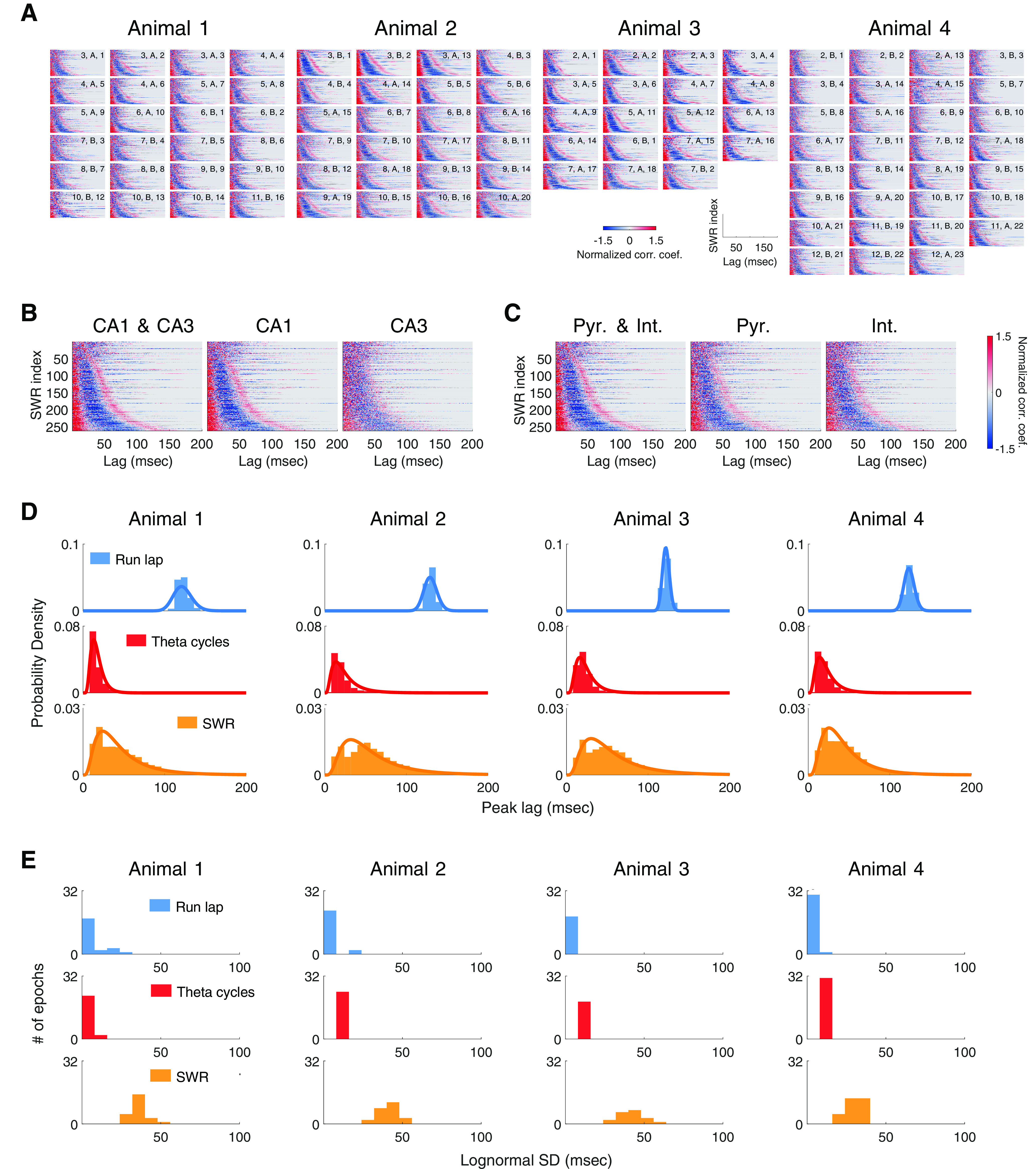
Wide distributions of periodicity for SWR-associated spiking. ***A***, Event-to-event variability in SWR-associated spiking organization across recording epochs, across days, and across animals. Each panel plots the histogram of the timing of the first positive side peak lag of the multiunit spiking autocorrelation for an epoch. The legend on top of each panel denotes the recording day, maze ID, and the exposure of the animal on the particular maze. For example, the legend “3, A, 1” means the panel plots the spiking activity from the third day of recording, during an exposure to track A that was the first such exposure. ***B***, ***C***, Similar patterns of SWR-associated rhythmic population activity in CA1 and CA3 and across putative excitatory (Pyr.) and putative inhibitory (Int.) cell types. SWRs within each panel are collated in ascending order by the timing of the first positive side peak lag. Every horizontal line is the autocorrelation function of an individual SWR; only non-negative lags are displayed. Positive and negative correlation coefficients are plotted in shades of red and blue, respectively. ***D***, Periodicity of SWR-associated spiking organization follows an approximate log-normal distribution in all four animals. Each panel plots the histogram of the timing of the first positive side peak lag of run lap (blue), theta-cycle (red), and SWR (yellow) autocorrelation function, for an example animal. Log-normal distribution fitted to the gamma range of each histogram is plotted as a solid line. ***E***, Histograms of the SDs of log-normal distribution fit to peak lag distributions across all epochs for an animal for run laps, theta cycles, and SWRs.

In contrast, the periodicity of the rhythmic SWR-associated population spiking spanned a wide and continuous range between ∼6 and ∼50 Hz (Fig. 2*B*, right). This continuous distribution was seen across the multiple run sessions within each day, across days, and across animals (Fig. 4*A*). The distribution was also visible in both CA1 and CA3, although the organization was clearer in CA1, perhaps because of the larger proportions of active neurons in CA1 compared with CA3 (Fig. 4*B*; Karlsson and Frank, 2008). The distribution was also visible across both putative excitatory and inhibitory cell types (Fig. 4*C*).

The periodicities underlying SWRs spanned a much wider range than those seen during run laps or theta cycles. To quantify this difference in spread, we fit parametric distributions to each empirical distribution (Fig. 4*D*). We found that the periodicities of SWR events were approximately log-normal, with the distribution median at the low end of the slow-gamma range: exp(μ) = 48 ms. Such a log-normal distribution creates a periodicity spectrum with a wide dynamic range, spanning from the majority of SWR events with a period between 25 and 75 ms to a small fraction of events with either a slower or a faster rhythmic organization. Importantly, the SWR-associated log-normal distributions had significantly larger SDs than locomotion-associated log-normal distributions (Fig. 4*E*; across all four animals, *p* < 10^– 242^, bootstrap tests).

**Figure 5.**
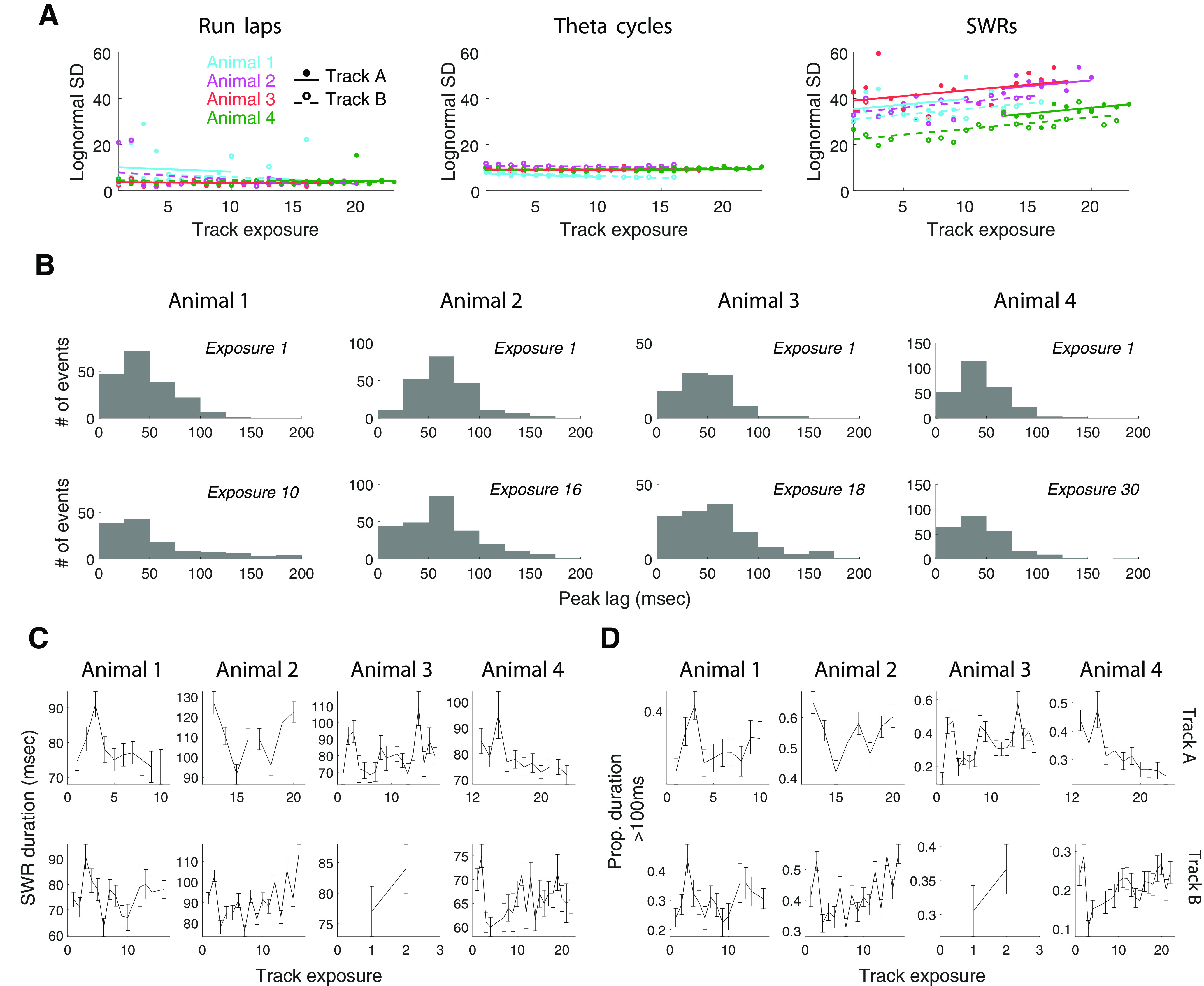
Increasing standard deviations of the log-normal distribution fit to SWR-associated spiking organization with track exposure. ***A***, Relationship between the number of exposures to a track environment and the SD of the log-normal distribution fit to peak lag distributions during run laps (left), theta cycles (middle), and SWR events (right). Each color denotes an animal. Each filled circle represents one epoch recorded in Track A, and each empty circles represents one epoch recorded in Track B. ***B***, Empirical distribution of SWR slow-gamma clock speed increased in spread between the first and last task exposure. SEM for mean by animal first versus last exposure: Animal 1, 1.99 versus 3.9; Animal 2, 1.91 versus 2.29; Animal 3, 2.53 versus 3.3; Animal 4, 1.42 versus 1.78. ***C***, SWR event duration across task exposures (median ± SE). ***D***, Proportion of long SWRs across task exposures (mean ± SE).

If this broad range of clock speeds serves an important function, it should be preserved throughout experiences in a given environment. Previous work has demonstrated that novel experiences drive high SWRs rates (O'Neill et al., 2006; Cheng and Frank, 2008), which then fall by about half as the environment becomes more familiar. To determine whether the range of clock speeds is preserved as the environment becomes more familiar and SWR rates decrease, we constructed a linear mixed-effects model (random intercept and random slope for exposure with animal-specific random effects). This model captures the modulation of the SD of the log-normal distribution by the number of exposures to each track environment, where exposure 1 means the first ever experience the rat has on a given maze [Fig. 5*A* (but see Fig. 5*B*, empirical distributions)].

Strikingly, we found that the SD that characterizes the distributional spread increased with familiarity. The SD was consistently and significantly positively correlated with the number of track exposures for SWRs (across four animals, coefficient = 0.50 and corresponding *p* = 4.91 × 10^–7^, *t* test for fixed effects), but not for locomotion-associated spiking events (across four animals, coefficient = –0.16 and corresponding *p* = 0.21 for run laps; and coefficient = –0.04 and corresponding *p* = 0.35 for theta cycles, *t* test for fixed effects; median of empirical SWR event durations and proportion of SWR duration >100 ms showed no consistent trend across the track environment or across animals; Fig. 5*C*,*D*). For visualization purposes, we plotted the regression lines for each track environment for each animal and observed that in the case of SWRs, they were approximately parallel to each other (Fig. 5*A*, right). This indicates a consistent change across environments and across animals, where the log-normal distribution of SWRs, but not locomotion-associated events, becomes broader as the environment becomes more familiar.

In our experiments, the rat experienced each environment across multiple days, and on each day there were multiple epochs. We therefore asked whether we could identify the periods when the distribution changed. Specifically, we asked whether there were detectable changes within a day, from one epoch to the next (consistent with within-epoch plasticity) and whether there were detectable changes across days, from the last epoch on day *n* to the first epoch on day *n* + 1, consistent with the plasticity during the ∼18 h of sleep and with the home cage experience between days. We used a simple linear mixed-effects model (random intercept with animal-specific random effects), applying a separate model for each environment. Since the overall distribution changes across days, we normalized the difference in the distributional spread to allow combining the data across days. We found that when comparing epochs within the same day, there is no statistically significant difference in the SD that characterizes the distributional spread (across four animals, intercept = –0.0077, *p* = 0.87, *t* test for fixed intercept), while when comparing the last epoch of a day with the first epoch of the next day, there is a statistically significant increase in distributional spread (across four animals, intercept = 0.12, *p* = 0.039, *t* test for fixed intercept). This suggests that the increase in the distributional spread across familiarity that we observed more likely occurred as a function of off-line plasticity outside of the maze.

#### The periodicity of SWR-associated rhythmic organization is correlated with LFP ripple power

**Figure 6.**
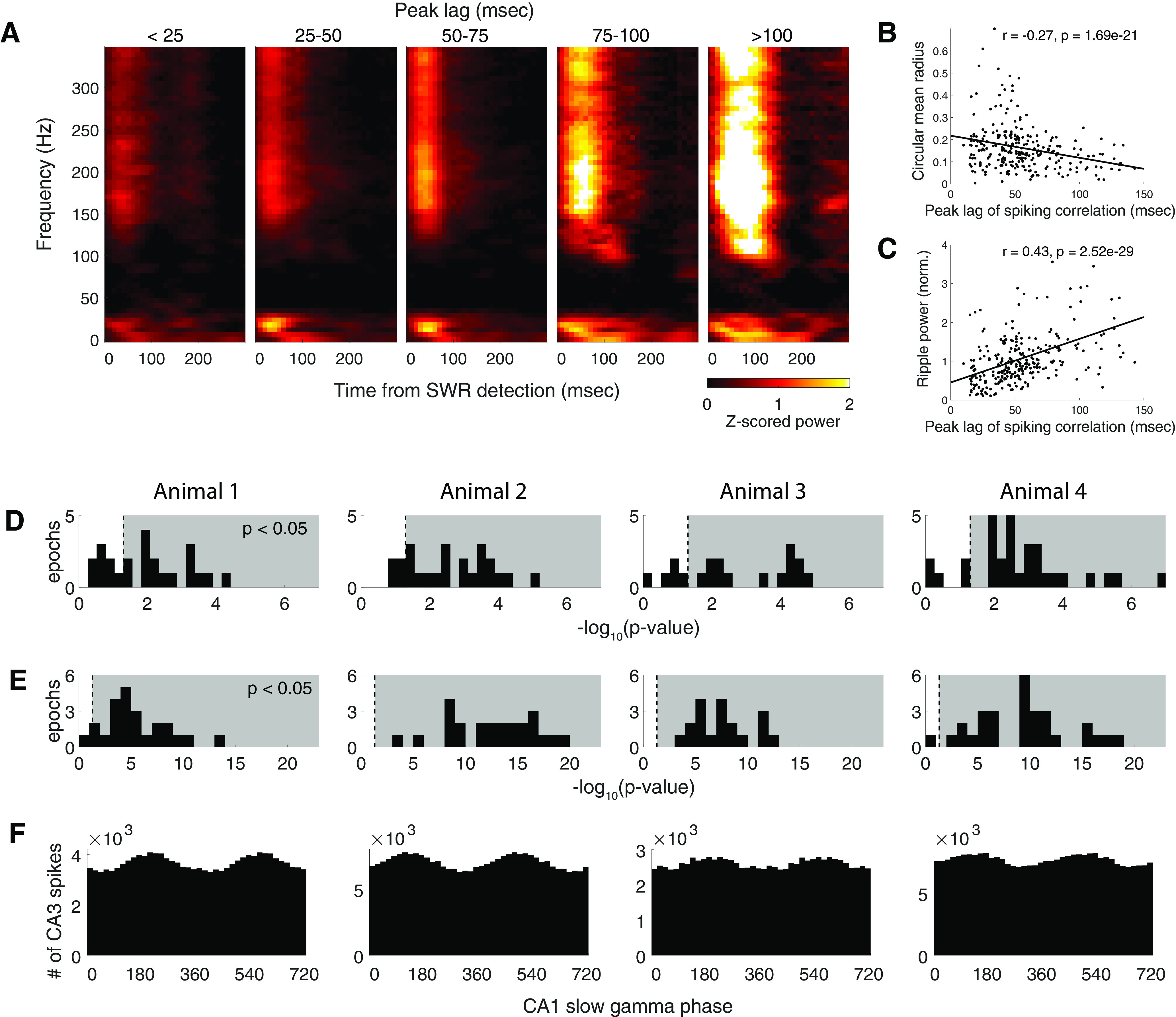
SWR-associated spiking organization correlates with the strength of gamma-phase locking and ripple power in the LFP. ***A***, Average SWR-triggered LFP spectrograms grouped by the first positive autocorrelation side peak (peak lag of autocorrelation) for an example epoch for Animal 2. ***B***, Slow-gamma phase locking (circular mean radius) MUA is negatively correlated with SWR peak lag for an example epoch for Animal 2. ***C***, Normalized ripple power is positively correlated with the peak lag for an example epoch for Animal 2. ***D***, Histogram, for each animal, of –log(*p*-value) for the Pearson correlation coefficient between the strength of the gamma phase locking of MUA and the peak lag of the SWR autocorrelation. Gray background indicates the area with *p* < 0.05. ***E***, Histogram, for each animal, of –log(*p*-value) for the Pearson correlation coefficient between normalized LFP ripple power and peak lag of the SWR autocorrelation. The gray background indicates the area with *p* < 0.05. ***F***, Spiking in CA3 is phase locked to slow-gamma oscillations in CA1. The histogram, for each animal, shows the numbers of CA3 multiunit spikes as a function of CA1 slow gamma phase.

How does this the periodicity of the SWR clock relate to the LFP? To address this question, we grouped all detected SWR events in a recording epoch into five categories based on the timing of the first positive peak lag of the SWR autocorrelation functions, from events with a fast rhythmic organization with periodicity <25 ms, to events with a stereotypical slow-gamma organization with periodicity between 25 and 50 ms, to events with a slow rhythmic organization with periodicity >100 ms. We then computed the average SWR-triggered spectrum of the LFP, normalized by the baseline spectrogram across an entire recording epoch (see Materials and Methods) for each of the five groups of SWR events (Fig. 6*A*). We observed that the strongest concentration of power in the slow-gamma band emerges in the groups whose autocorrelation lags have peaks between 25 and 75 ms. This concentration of power gradually shifts to lower frequencies as the peak lag of the SWR autocorrelation increases. We also observed increased power at ripple band as the peak lag of the autocorrelation increases.

We then quantified these relationships. We first measured the strength of the slow-gamma phase locking of the multiunit spiking activity and confirmed that across all lags, there was significant phase locking to slow gamma (*t* test, *p* = 3.6 × 10^–73^; across all four animals, *t* tests for 100% of epochs, *p* < 0.001). We also observed that slow-gamma phase locking decreased as the clock speed increased [Pearson's *r* = −0.27, *p* = 1.69 × 10^– 21^, *n* = 262 pairs; Fig. 6*B* (but see Fig. 6*D*, analyses across all four animals)]. Importantly, this phase locking was also present for CA3 spikes alone (across all four animals: *p* $=5.502\times 10^{-76},4.901\times 10^{-142},1.828\times 10^{-21},9.787\times 10^{-72}$; Rayleigh's test for unimodality; Fig. 6*F*), as expected given previous results (Carr et al., 2012). In contrast, there was a significant inverse relationship between ripple power and clock speed, with lower normalized ripple power at higher clock speed [Pearson's *r* = 0.43, *p* = 2.52 × 10^–29^, *n* = 262 pairs; Fig. 6*C* (but see Fig. 6*E*, analyses across all four animals)].

**Figure 7.**
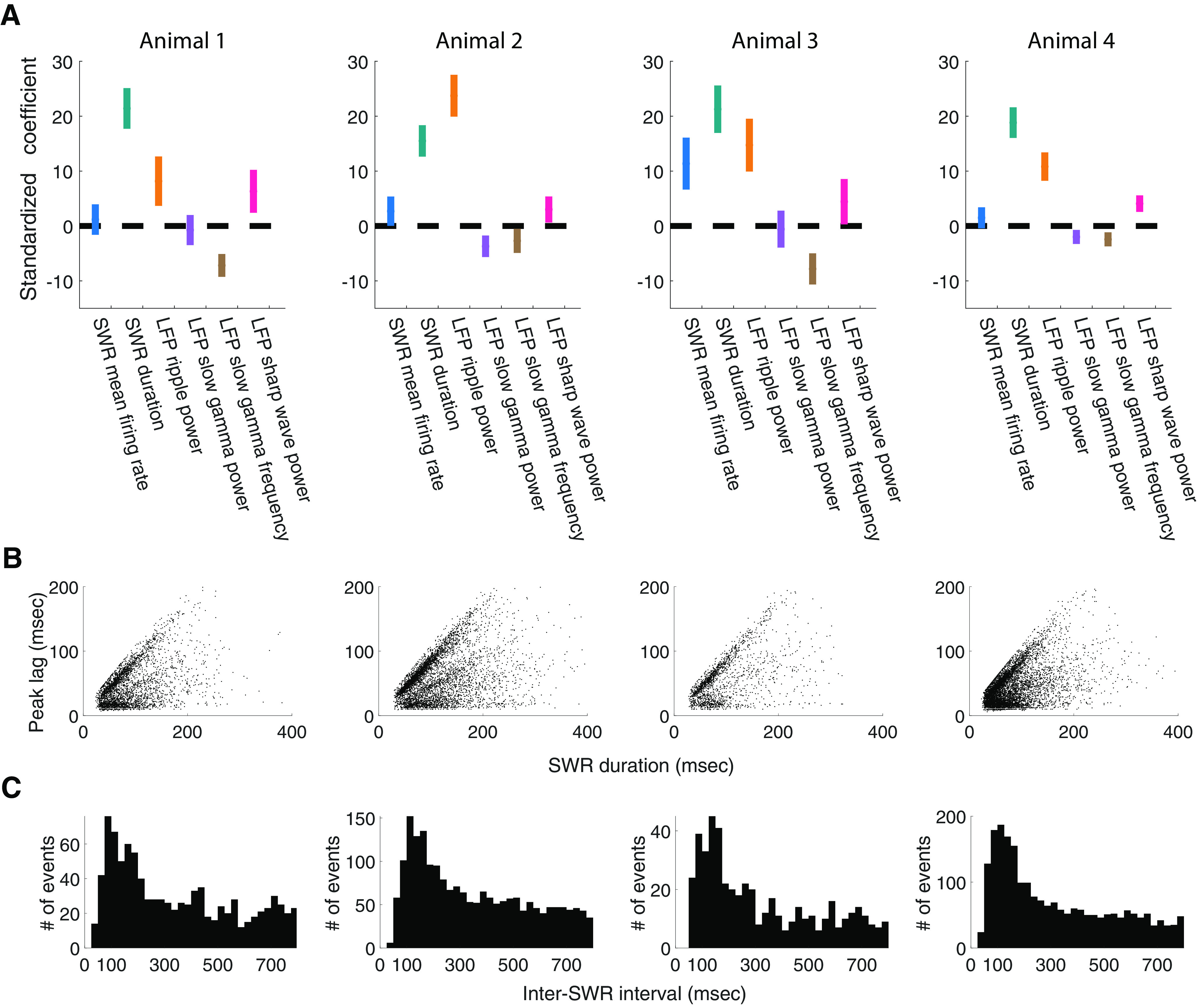
Periodicity of SWR-associated spiking organization is correlated with LFP ripple power and event duration. ***A***, Regression coefficients and confidence intervals for six SWR-associated covariates as predictors for peak lag for each animal. Vertical line segments indicate 95% confidence intervals of the standardized coefficients for fixed effects of event mean firing rate (plotted in blue), event duration (green), LFP ripple power (yellow), LFP slow-gamma power (purple), LFP slow-gamma frequency (brown), and LFP sharp-wave power (red) in a multiple linear mixed-effects regression model with random effects for epochs. Across animals, the duration of SWR events and LFP ripple power were significantly positively correlated with the peak lag of the spike autocorrelation, while LFP slow-gamma frequency was significantly negatively correlated with the peak lag of the spike autocorrelation. ***B***, Periodicity of SWR-associated spiking organization versus event duration. Note the density of events near the diagonal (*x* = *y*) line reflecting events with a peak lag only slightly shorter than the event itself. Also note that a small number of points (*n* = 177, 126, 65, and 438 for animals 1-4, respectively) are above the *x* = *y* line. These values are a result of the Gaussian smoothing of the autocorrelations, which can occasionally result in autocorrelation mass outside the event duration. ***C***, Empirical distribution of inter-SWR interval clusters around the theta frequency (∼120 ms).

These initial analyses identified covariates of the clock speed, and motivated a more comprehensive model. We therefore constructed a multiple linear mixed-effects regression model with random effects of epochs that captured the modulation of the SWR-associated spiking organization with a set of parameters defining the influence of the mean population spiking rate, SWR event duration, LFP ripple power, LFP slow-gamma power, and LFP slow-gamma peak frequency (Fig. 7*A*). We estimated the values of these parameters that maximize the likelihood of the observed SWR population spiking. Here we focus on the parameters that were significant predictors across all four animals.

We found that LFP slow-gamma frequency was significantly negatively correlated with the peak lag of the autocorrelation (across all epochs for four animals: coefficient = –7.19, –2.70, –7.82, –2.44; corresponding *p* = 1.57 × 10^– 11^, 0.016, 6.80 × 10^– 8^, 1.78 × 10^– 4^, respectively; *t* tests for fixed effects), which is consistent with the qualitative observations from the previous spectrogram analysis (Fig. 6*A*). The regression analysis further showed that, after accounting for the effects of other SWR temporal and frequency components, LFP ripple power remained significantly positively correlated with a peak lag of (across epochs for four animals: coefficient = 8.16, 23.72, 14.73, 10.81; corresponding *p* $=3.83\times 10^{-4},6.89\times 10^{-34},2.33\times 10^{-9},2.63\times 10^{-16}$, respectively; *t* tests for fixed effects).

The duration of individual SWR events was also significantly positively correlated with the peak lag (across all epochs for four animals: coefficient = 21.41, 15.49, 21.27, 18.82; corresponding *p* $=2.582\times 10^{-29},4.163\times 10^{-26},1.575\times 10^{-21},1.606\times 10^{-39}$, respectively; *t* tests for fixed effects). Thus, as one might expect, the slower the hippocampal rhythmic organization underlying an individual SWR event is, the longer the duration of the event. An SWR cannot have a peak lag longer than its duration, and indeed many SWRs had a peak lag only slightly shorter than their duration (Fig. 7*B*). We therefore repeated the quantification of the correlation considering only events >100 ms. This yielded the following mixed results: for one animal, event duration was no longer significant (*p* = 0.88), but for the other three animals, event duration was still a significant predictor at the α level of ≤0.1 (*p* = 0.0064, 0.086, 0.0013, respectively; *t* tests for fixed effects).

The peak lags of SWRs ranged from ∼25 to ∼125 ms with the most common values near 50 ms. To determine how the distribution of peak lags we measured related to the typical intervals between SWRs, we plotted the empirical distribution of inter-SWR intervals (Fig. 7*C*). We found that, consistent with previous experimental observations (Yamamoto and Tonegawa, 2017), the time between SWRs was typically >100 ms, and this empirical distribution clusters around theta frequency (∼120 ms) and its harmonics. This period is much longer that the period of the internal SWR clock, suggesting that a different process governs intra-SWR versus inter-SWR activity.

#### Periodicity of SWR-associated rhythmic organization is correlated with classifiability, but not types of sequential representational content

**Figure 8.**
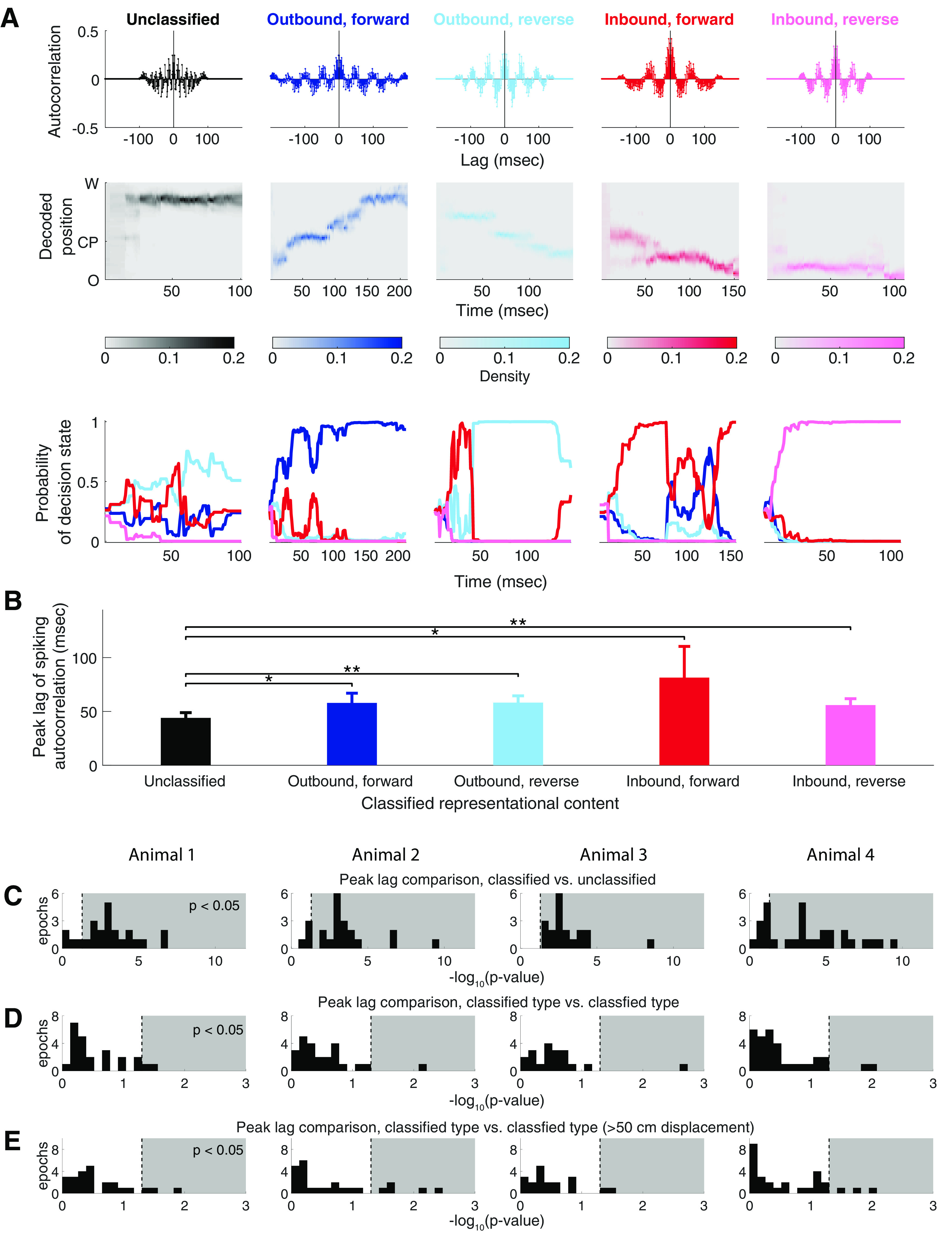
SWR-associated spiking organization correlates with classifiability of representational content, but not the type of the content. ***A***, Examples of SWR events with different classifications of representational content. Each column represents one SWR. Top, Autocorrelation function of population spiking. Middle, Decoded trajectory where the heat plot shows the estimated posterior density at each time step. Bottom, Probability of the decision state as a function of time. Probability of the SWR event representing an outbound, forward path, an outbound, reverse path, an inbound, forward path, or an inbound, reverse path is plotted in darker blue, lighter blue, darker red, and lighter red, respectively. ***B***, Peak lags of the SWR autocorrelation function grouped by classification of representational content for an example epoch of Animal 2. The group of SWR events whose decision-state probabilities did not pass the threshold and cannot be classified is plotted in gray. The groups of SWR events whose representational content can be classified with confidence as an outbound, forward path, an outbound, reverse path, an inbound, forward path, or an inbound, reverse path are plotted in darker blue, lighter blue, darker red, and lighter red, respectively. All bar graphs show the mean + 1.96 × SEM. Lines and asterisks indicating significant differences between pairs of groups under Wilcoxon rank-sum one-sided tests with Bonferroni correction. *p-value < 0.05; **p-value < 0.01. ***C***, Histogram, for each animal, of the *p*-value of Wilcoxon rank-sum tests for an ordinal difference between peak lags of the spiking autocorrelations of the unclassified events and that of the classified events across epochs. ***D***, Histogram, for each animal, of *p*-values of Kruskal–Wallis tests for a difference between peak lags of the spiking autocorrelations across groups with different types of representational content among classified events across epochs. ***E***, Histogram, for each animal, of *p*-value of Wilcoxon rank-sum tests for an ordinal difference between peak lags of the spiking autocorrelations of classifiable events with well defined trajectory (decoded position displacement, >50 cm) and those of classifiable events that do not have a well defined trajectory.

Finally, we asked whether the rhythmic spiking organization of an SWR event relates to its representational content. We applied a previously developed and validated discrete decision state point process filter (see Materials and Methods; Deng et al., 2016) to classify the representational content of SWR events from multiunit spiking activity into four categories (an outbound, forward path; an outbound, reverse path; an inbound, forward path; or an inbound, reverse path), based on the direction and temporal order of the spatial trajectory and computed our confidence about the classification. Events whose decision state probabilities did not pass the confidence threshold and cannot be classified into any of the four aforementioned categories are denoted as unclassified (Fig. 8*A*, example SWRs). When we examined all SWRs, we found there was a statistically significant difference between the peak lag of the spiking autocorrelation of the unclassified group and that of each of the classified groups [Fig. 8*B*, example epoch: Wilcoxon rank-sum one-sided tests with Bonferroni correction, *p*(unclassified vs outbound, forward) = 0.035, *p*(unclassified vs outbound, reverse) = 0.008, *p*(unclassified vs inbound, reverse) = 0.021, and *p*(unclassified vs inbound, reverse) = 0.0027, for analyses across all four animals; Fig. 8*C*]. This result suggests that SWRs with unclassified content (i.e., SWRs that do not represent a trajectory through space) tend to have a faster rhythmic organization, while SWRs with classifiable content tend to have a slower one. We then examined only SWRs with classifiable content and found no statistically significant differences (Kruskal–Wallis tests; for analyses across all four animals; Fig. 8*D*). The same was true when we restricted the analyses to classified events and compared those that contained extended trajectories (decoded position displacement, >50 cm) with those that did not: there was no statistically significant difference in peak lags (Fig. 8*E*). Thus, among SWRs with classifiable content consistent with representations of trajectories, there is no correlation between the periodicity of rhythmic spiking organization and the type of trajectory-related spatial content.

## Discussion

We explored the structure of spiking activity across periods of locomotion and during hippocampal SWR events. We found that the periodicity of SWR-associated hippocampal rhythmic spiking has much higher event-to-event variability than that of locomotion-associated spiking. This SWR-associated clocking variability was observed across recording epochs, experiment days, and animals. The wide range of periodicities (e.g., the many different periods of the SWR clock) was clearly visible in novel environments where SWRs are very prevalent (O'Neill et al., 2006; Cheng and Frank, 2008) and actually increased in more familiar environments. No such changes were present for locomotion-associated spiking. The large variability in the spiking organization within SWRs, combined with the observation that the brain can maintain less variable timing during locomotion, demonstrates that lower variability is within the capacities of the system and suggests that the higher variability seen during SWRs serves a function.

### SWR periodicity, slow gamma, and theta

We found that the periodicity of the clock within theta cycles seen during locomotion has low variability and clusters around the fast-gamma range. This is consistent with two recent studies that have investigated hippocampal subsecond dynamics during locomotion on a cycle-by-cycle basis using frequency domain tools (Lopes-Dos-Santos et al., 2018; Zhang et al., 2019). Zhang et al. (2019) analyzed cycle-to-cycle changes during locomotion theta oscillations using frequency decomposition methods on LFP recordings, and found that fast-gamma states were dominant. Lopes-Dos-Santos et al. (2018) designed an unsupervised framework to extract the spectral content of individual theta cycles and found that “theta-nested spectral components were differentially altered by behavioral stages of a memory task; the 80 Hz mid-gamma component was strengthened during learning, whereas the 22 Hz beta, 35 Hz slow-gamma, and 54 Hz mid-gamma components increased during retrieval. These four components correspond to peak lags of 12.5, 45.5, 28.6, and 18.5 ms, all of which were present in the empirical distribution of peak lags in our analyses for theta cycles, although not with uniform frequency, as follows: 12.5 ms (80 Hz) and 18.5 ms (54 Hz) peak lags are most frequently observed in theta cycles in our datasets; 28.6 ms (35 Hz) peak lags are observed relatively less frequently; and 45.5 ms (22 Hz) peak lags are observed the least frequently. Nonetheless, as all four frequency ranges were observed, and as we did not attempt to separate learning from retrieval, our results should not be seen as contradicting those of Lopes-Dos-Santos et al. (2018).

We also found that the periodicity of this clock during awake immobility is related to the frequency of the slow-gamma rhythm in each SWR. This is consistent with prior work (Carr et al., 2012; Pfeiffer and Foster, 2015) and also with the results presented in a recent report from Oliva et al. (2018), although those authors came to a different conclusion about the data. They claimed that the slow-gamma power increase underlying SWRs is a “spurious” oscillation because “slow gamma power is specifically associated with longer SPW-Rs [sharp-wave/ripple complexes] produced by the overlap of multiple ripple events” (Oliva et al., 2018). This conclusion was partially based on a replication of the observation of coupling between slow-gamma phase and ripple amplitude (Carr et al., 2012), indicating bursts of ripple power at slow-gamma frequency. The authors did not present a definition of an actual oscillation that would allow for a clear separation from spurious oscillations, however, making it difficult to evaluate their conclusion. Their conclusion is also puzzling given the well established presence of slow gamma as a reflection of CA3 input to CA1 (Colgin et al., 2009; Kemere et al., 2013), the locking of CA3 and CA1 spikes to slow gamma during SWRs (Carr et al., 2012), and the known role of CA3 input in driving SWRs in CA1 (Buzsáki, 2015).

We suggest instead that the question should be whether an oscillation extracted from the wideband LFP reflects a real physiological process (in this case, CA3 input to CA1) and is useful in understanding information processing in the brain. Using that criterion, our findings corroborate previous work (Carr et al., 2012; Pfeiffer and Foster, 2015) and indicate that measuring power and phase in the slow gamma provides important information about SWR characteristics, including the presence of sequential content (Carr et al., 2012). We found that, despite the wide range of clock speeds resulting in different numbers of clock cycles for each individual SWR event, multiunit spiking in CA3 was significantly phase clocked to the slow-gamma clock in CA1. We further showed that, even after accounting for event duration, mean firing rate, and other local field frequency components, the periodicity of this slower, rhythmic spiking process that underlies SWR events remains significantly correlated with LFP ripple power, which relates to pyramidal cell synchrony (Csicsvari et al., 2000). These findings strengthen the association between the temporal structure of SWRs and the robustness of spiking activity within the events, suggesting that modulation in a slow-gamma range facilitates the expression of longer SWRs. At the same time, we identified a small number of events with a clock as slow as ∼6 Hz. This suggests that the slow-gamma range may better be defined as a log-normal distribution that extends to lower and higher frequencies.

We additionally found that the interval between SWRs was most often >100 ms. Consistent with prior work (Yamamoto and Tonegawa, 2017), this empirical distribution of inter-SWR intervals peaked near the period of the theta rhythm (∼125 ms) with some suggestion of peaks at harmonics of theta. Thus, during awake immobility, SWRs and their associated slow-gamma periodicity might ride on top of low-amplitude theta oscillations. Theta–gamma coupling has long been observed occurring together in the hippocampal circuits during locomotion (Colgin et al., 2009). Our results here, together with previous experimental and theoretical work, suggest that theta and slow-gamma oscillations provide a more general organization scheme for learning and memory in the hippocampal circuits, both during locomotion and during awake immobility (Lisman, 2005; Lisman and Buzsáki, 2008; Lisman and Jensen, 2013).

### A changing hippocampal clock is a potential mechanism for flexible memory retrieval

Is there a possible functional role of the SWR-associated rhythmic organization and of its variability? There are two possibilities. One is that the brain wishes to impose a uniform temporal structure when storing or updating representations, but, because of intrinsic variability (Atallah and Scanziani, 2009; Hemberger et al., 2019), it fails to do so and produces variably timed replay events. The other possibility is that replaying the same spatial content with varying degrees of rhythmic organization is beneficial for flexible storage. In this scenario, the goal would be to store memories with a more flexible temporal structure that would provide for different degrees of “chunking” in the hippocampus and, presumably, in downstream cortical networks that are engaged during SWRs (Buzsáki et al., 2013).

Our data are more consistent with the second of these two possibilities. We found that the hippocampus can express sequences with very low temporal variability, both across and within theta cycles seen during locomotion. This indicates that the hippocampus can generate spiking patterns that are tightly locked to a given frequency. By contrast, the temporal variability of SWR-related spiking is high during new experiences and increases as the environment becomes more familiar. In other words, as an animal learns the task in an environment, hippocampal circuits replay spatial representations with an underlying internal periodicity (clock) sampled from an increasingly broad log-normal distribution. We note that, in our dataset, this is not associated with a consistent experience-dependent trend for its median or tail probability (proportion of an event >100 ms; Fernández-Ruiz et al., 2019).

This increasing variability indicates that the variability in the clock is not solely a result of instability associated with recent learning and suggests that the variability could be a feature rather than a bug. Indeed, individual experiences could be replayed with very different temporal organization, and that the identifiability of SWRs as content-full representations was similar across a wide range of clocks. At the same time, events with a shorter period tended to be less classifiable using out model, but even these events could serve a purpose. Here we defined classifiable events as those that contain sequential activation of adjacent spatial elements. Recent work from our group has expanded this definition to include events that can include stationary dynamics (e.g., representations of a place or a small snippet of a trajectory) as well as fragmented dynamics (e.g., spatially disorganized representations; Denovellis et al., 2021). We therefore suggest that the more nonclassifiable events could serve to create either more focused associations relevant to a particular location or more diffuse associations across nonadjacent locations that could be useful to grouping experiences that occur within the same overall context. This would further suggest that SWR-associated replay is not a simple, uniform compression of experience, but rather an instantaneous, random sample (Foster, 2017; Stella et al., 2019), and such a random sampling scheme allows for an unbiased, efficient representation of experience. This sampling hypothesis is also consistent with a recent study that showed hippocampal replay can represent Brownian diffusion-like random trajectories that cover positions over wide ranges of spatiotemporal scales (Stella et al., 2019).

More broadly, human cognition is characterized by its extreme flexibility—the ability to transfer past learning to new contexts and to form abstract thoughts, such as analogies and inferences, to guide behaviors (Behrens et al., 2018). One crucial prerequisite of this flexibility is the ability to remember past experiences at different levels of specificity. In particular, advances in reinforcement learning showed that there are advantages to learning and memory when past experience is represented at different levels of temporal abstraction (Schmidhuber, 1992; Sutton et al., 1999; Bakker and Schmidhuber, 2004; Kulkarni et al., 2016; Konidaris, 2019; Levy et al., 2019). More recently, a human imaging study observed that speed of time-compressed forward replay flexibly changes in human episodic memory (Michelmann et al., 2019). Our observations suggest that the hippocampal circuits replay spatial experiences at multiple levels on an event-to-event basis during SWRs. We propose that such a variable clock might constitute a general mechanism for flexible memory storage that could enable subsequent flexibility in behavioral choices.
